# The Laws of Health, in Relation to Mind and Body; a Series of Letters from an Old Practitioner to a Patient

**Published:** 1851-11

**Authors:** 


Art. VIII.—*The Laws of Health, in relation to Mind and Body: a series of Letters from an old Practitioner to a Patient.* By Lionel John Beale, M.R.C.S. Philadelphia: Blanchard & Lea. 1851. 8vo. pp. 306.

We should have supposed it scarcely possible that any one could invest, what many of the profession no doubt consider a hacknied theme, with a fascination that knows no fatigue, but Mr. Beale has earned just such a triumph in the eloquent and instructive work before us. His book was undoubtedly designed mainly for popular reading, but we grieve to say that much of what Mr. Beale considers well known to the profession, is scarcely any better known to its members, than to the hangers on of general society. The author's powerful and forcible appeals to society on the vast necessity of attention to sanitary laws, should be directed to the profession, for it is the profession of medicine that needs reform in these matters. We have some experience in sanitary matters, and where we have found mole-hills in the populace, we have met with mountains in the medical profession. The great work of reform must commence with the medical profession. Let its members right themselves in sanitary science, and society will be no laggard in working changes. It is strange, that after the fiat of Omnipotence gave to man dominion over the earth, he has permitted it to be his conqueror; that from its bosom he suffers the destroying pestilence to spring, to waste empires in its fervors, and to undermine society in its more slothful vocations. The air that should give vigor, health, strength, and joy is tainted with noxious powers that transform the atmosphere of life to one of death. The water that gushes from its mountain home, cool, sparkling, and pleasant to the eye, contains the seeds of sickness and decay. The highest element of mind is that which looks best and farthest into futurity; that, which not content with the evanescent enjoyments of the present, seeks the substantial fruits that conduct most securely deep into the future.

Schools are provided by the State for the education of the young; it is assumed that the Commonwealth is bound to furnish its rising generations with capacity at least to read. And is it not the duty equally of the State to pay some attention to what is read? Is it a matter of indifference whether children shall read "The Three Spaniards," "the Mysteries of Udolpho," "Abellino, the Bravo of Venice," or some good treatise on Physiology? If children are trained in supreme ignorance of the laws of life, is it likely that society will be quickened into vital truths on the subject? It is shameful that in the public schools of Kentucky, those schools with which it has pleased the majesty of the government of the Commonwealth to connect itself, exactly in the same way the same sovereignty connected itself with the construction of turnpike roads, we say is it not shameful that the study of the laws of life, the acquaintance with the laws of respiration, of digestion, of bodily cleanliness, and of the relations of sentient beings with surrounding nature, are as sedulously avoided as are the mysteries of the Chinese language? So profound is the ignorance, so universal the taint, so apathetic the public and professional mind on this all-important department of knowledge, that is essential during each moment of life, that we doubt whether the energy, the eloquence, and devotion even of Robert J. Breckinridge can breathe the breath of life into the almost hopeless inanimation that surrounds him. How many hopes, how many future prospects, how much of the well-being of the State hang suspended on this theme! We should rejoice to be able to record among the brilliant services of the Superintendent of Public Instruction, that though he found all physiological instruction in the public schools of Kentucky a blank, he left it among the flourishing realities of elementary teaching. But we must trust our pen no further. now, on this absorbing theme.

Mr. Beale's epistolary labors are divided into twenty-eight chapters, and they comprise the very best knowledge we have ever seen on the subjects to which the work is devoted. The first chapter is dedicated to a general and concise view of the importance of the themes to which the book is devoted; the second is a noble tribute to the medical profession, and to the great names that have adorned it; the third is a castigation of quackery in all its forms. The next four chapters are devoted to the elucidation of the physiological laws of digestion, respiration, the skin and its functions, and the brain and its functions. Nine letters are occupied with the phrenological doctrines, and neither Gall, Spurzheim, Combe, nor Caldwell has presented the truths of that science more urgently and attractively. If every parent and preceptor in the land had their whole conduct imbued with the profound truths developed by Mr. Beale in the letters commencing at the eighth, and terminating with the sixteenth, we should have great hopes of human nature.

The remaining letters are occupied with the changes incident to the various stages of life, and more invaluable knowledge, of a temporal character, never was comprised within the same space.

Medical men seem to feel that a portion of their mission is war upon quackery in all its multitudinous forms. If they would win a triumph let them apply the axe to the root of the tree, for it is through ignorance that quackery flourishes. We fully agree with Mr. Beale in the following sentiments:

"It is ignorance that affords patronage to secret remedeis, miraculous cures, and quackeries of all sorts, both in and out of the domain of physic. When all medical practitioners shall cease to be pretenders to more knowledge than they really possess, then will the public cease to patronize quackery; and a more complete education—intellectual, moral, and professional—of all classes of medical practitioners, engendering higher views of their duties, will cause them to rank higher in the estimation of the public, and be productive of greater benefit, than any exclusive privileges which the Legislature could confer upon them."

We regret that want of space forbids much display of quotation. We had marked numerous passages for comment, and now, when want of space restricts us to narrow limits, it is difficult to say what shall be chosen. There are many of the elements, not only of the prevention, but the cure of disease in the following remarks:

"Full expansion of the chest is equally essential to health as good air, for if by our clothing or constrained position, we impede the full expansion of the lungs, healthy respiration is prevented, and the due purification of the blood impaired. Whatever compresses the chest or abdomen impedes respiration, and therefore, pressure from dress, bands, or stays, must always be bad. How is the chest of a girl to expand with growth if incased in these horrid inventions? No girl should wear stays till she has long done growing, for the chest continues to expand after growth has ceased: by the use of stays the size of the chest is limited, and the ribs are forced to overlap, as I have seen in several instances. I question if any woman would really require stays before the age of thirty-five or forty. The best figures of ancient and modern times have never worn any stays. We have dismissed the swaddling clothes of our infants, and we shall succeed, sooner or later, in annihilating stays for girls and young women. None should wear them if they knew how much better they would be without. After having been accustomed to the support it is very difficult to discontinue their use, because the muscles of the spine, having been superceded in their action by the barbarous pieces of iron, bone, or wood of these body-cases, have lost their power of maintaining the body in an upright position, and without stays the deformities produced by these machines become visible. I hope the time will arrive when stays will be considered antiquities of the mediæval ages, and be only preserved as relics to adorn the museums and halls of the curious.

"It is a law of health that we should every day be in a position to empty the whole of the lungs, and by rapid and frequent dilitation of the chest change the air they may contain; hence the benefit of strong and active exercise; boys do this for themselves in their games, but the dreaming walk of a girl's school produces no such beneficial influences. Exercise is as necessary for the development and health of the lung, as it is for all the other organs, therefore singing and declamation are beneficial. It has been observed by an ingenious writer, that exercise not only ameliorates living matter but dead matter also, violins, flutes, and other musical instruments improving by use. * * * * The appetite and spirits produced by mountain air arise from the great activity imparted to the breathing from climbing, and to the mind from frequent change of scenery. Residence in such a district imparts to the whole population elasticity of mind and vigor of body, and we should naturally expect the formation of a national character totally different from that induced by the monotony of a flat country."

Here, however, we must stop; but we beg our readers not to fail to adorn their minds with the living and eternal truths of this little volume. It is replete with interest and instruction, and cannot fail to improve all who may study its important matters. We acknowledge ouiself greatly the debtor to Mr. Beale, not only for the cogent manner in which he urges truths with which we were familiar, but for an increase of information.

These works may be found at the bookstore of Beckwith & Morton, who are always prepared with a general assortment of medical books. B.

---

Want of space prevents an analysis of De la Beche's great work on Geology, Walshe on the Heart and Lungs, Wythes' work on the Microscope, and Walker on Intermarriage. We shall endeavor to make our readers familiar with the contents of these volumes in our next number. B.

